# Lockdown through a Chinese lens: A qualitative study

**DOI:** 10.1177/13634615241296310

**Published:** 2025-01-29

**Authors:** Doris Zhang, Gary Cheung, Sarah Cullum, Lillian Ng

**Affiliations:** 1415 Department of Psychological Medicine, Faculty of Medical and Health Sciences, The University of Auckland, Auckland, New Zealand

**Keywords:** Chinese culture, residential facilities, social isolation, older adult, COVID-19 lockdown, psychiatry

## Abstract

COVID-19-related lockdowns resulted in strict visiting restrictions in care homes, placing a vulnerable population at further risk of functional and cognitive decline, and psychological difficulties due to isolation. Experiences of vulnerable minority groups of older persons who reside in care homes are not well researched. In New Zealand, the Chinese community is a fast-growing ethnic group that faces challenges such as language barriers, differing cultural beliefs and COVID-19-related discrimination. The aim of this study was to explore the experiences of Chinese care home residents in New Zealand during COVID-19 lockdowns. In this qualitative study, we interviewed residents (*n* = 6), family members (*n* = 6) and facility staff (*n* = 6) across two Chinese-run care homes in Auckland, New Zealand. Resident and family member participants were exclusively Chinese. Interviews were conducted and transcribed in either English or Mandarin Chinese. Transcripts were coded and analysed to synthesise themes. We identified five themes: (a) acceptance and pragmatism; (b) attitudes towards authority; (c) the concept of *máfan*: 麻烦 (to trouble); (d) challenges to fulfilling filial duties; and (e) responding to pandemic challenges. This research reframes the narrative of older Chinese care home residents during COVID-19-related restrictions. We recommend integrating the findings and philosophical values identified in this study to develop future protocols that consider the cultural and language needs of Chinese care home residents.

## Introduction

The COVID-19 pandemic had a dramatic impact on all sectors of society, particularly the older age group. New Zealand is an ageing society, with the proportion of people aged 65 years and above projected to increase by approximately one-third in the next 30 years from 16.9% in 2023 to 23.8% in 2053 (Statistics New Zealand, 2021b). The older population (age 65+) of ethnic minority groups are projected to increase at a higher rate than their New Zealand European counterparts, particularly Asian subgroups (Office for Seniors, 2020). The circumstances of a pandemic give rise to questions about how care home residents experience lockdown conditions.

The Asian ethnic group in New Zealand encompasses individuals who self-identify with origins in East, South East and South Asia. In 2018, the Asian ethnic group was the fastest growing and third largest ethnic group in the country (Statistics New Zealand, 2018b). The largest subgroup was Chinese, comprising approximately one-third of the total Asian population (Statistics New Zealand, 2018a). The diasporas are culturally diverse and mostly originate from Mainland China, followed by Taiwan and Malaysia (Liao, 2007). Often, older Chinese emigrate to New Zealand to reunite with their children and to assist with raising grandchildren (Yeung & Allen, 2020).

There has been steady growth in older adults choosing to reside in aged residential care (ARC) in recent years. In 2018, there were ∼38,600 beds operated by 668 facilities in New Zealand, with a relatively high per capita use by the over-65 population compared with other OECD countries (Ernst and Young, 2019; McDougall, 2018). Nearly half of those aged 65 and above will use ARCs at some point in their lives (Broad et al., 2015). The total ARC cost attributable to dementia in New Zealand is $1.21 billion (Ma’u et al., 2021). Half of all care home residents are diagnosed with dementia – a number likely to increase because of an ageing population (Central Region Technical Advisory Services Limited, 2019). The prevalence of dementia in the Asian ethnic group is projected to double from 6.9% to 13.3% by 2040 (Ma’u et al., 2021). There is a lack of culturally relevant services for older Chinese who reside outside Asia (Cheung et al., 2019; Mukadam et al., 2011; Roche et al., 2021).

The high burden of dementia makes it challenging for care homes to deliver services, particularly because of a lack of funding and staffing shortages (Dudman et al., 2018; Kiata et al., 2005). Internationally, the COVID-19 pandemic had a disproportionately negative impact on older people who reside in care homes (Thompson et al., 2020). Care home residents were at higher risk of SARS-CoV2 infection and mortality because of pre-existing comorbidities (Broad et al., 2020; Shahid et al., 2020). Care home residents were over-represented in total COVID-19 deaths (Comas-Herrera et al., 2020).

Lockdowns resulted in feelings of entrapment and disconnection from the outside world, preventing residents from maintaining social connections, which are crucial for mitigating loneliness (Ayalon & Avidor, 2021; Chee, 2020). Infection control measures were associated with psychological and physical harm owing to subsequent social isolation and immobility (Gordon et al., 2020). Visiting restrictions led to further isolation from family carers, and isolating residents in their rooms away from communal areas resulted in difficulties in maintaining safe staffing (Gordon et al., 2020).

Auckland is New Zealand's largest city and comprises 1.7 million people. It is the located on the North Island of New Zealand, and has a multicultural population: 53.3% identify as New Zealand European, 28.2% as Asian, 11.5% as Māori and 15.5% as Pacific Peoples (Statistics New Zealand, 2019, 2021a, 2021b). In the 2018 census, 70% of the total New Zealand Chinese population resided in Auckland (Statistics New Zealand, 2018a, 2018b). On 25 March 2020, New Zealand entered a nationwide lockdown in response to the COVID-19 pandemic. There were three cycles of lockdowns from March to June 2020, August to October 2020 and February to March 2021, with some restrictions limited to the Auckland region. These lockdowns were guided by a four-tiered alert level system, with level 4 being the most stringent. This study was undertaken during a time at which there were no restrictions in Auckland from 21 April to 6 June 2021, which allowed for in-person interviewing in care homes.

In New Zealand, Chinese-run care homes are in the minority, with most care homes catering to mainly English-speaking residents. There has been increased demand for care homes that cater to residents that preferentially speak Cantonese or Mandarin. These care homes also have Chinese-style food and celebrate Chinese cultural events. We selected Chinese-run care homes to provide a uniquely Chinese perspective on the lockdown experience. Residents in these care homes may face barriers of language and culture, as identified among New Zealand Chinese more generally (Yeung & Allen, 2020).

### Aim

Cultural and language barriers may place Chinese care home residents at increased risk of social isolation during lockdown periods. They may also be subject to discrimination, because the coronavirus originated in Wuhan, China (Nielsen, 2021; Yeung & Allen, 2020). This study aimed to explore the experiences of Chinese care home residents living in New Zealand during three lockdown periods from 2020 to mid-2021. We sought to understand how Chinese care home residents lived through the lockdown conditions and how their care needs could be better met in future pandemics.

## Methods

### Ethics and recruitment

Ethics approval was obtained from the Auckland Health Research Ethics Committee (reference number: AH1384). The research team contacted the care home managers of two Chinese-run care homes in Auckland in early April 2021. These two facilities were purposively chosen, because the managers were previously known to the research team. An invitation email, participant information sheets and consent forms were sent through email or WeChat, a widely used Chinese social media application, for circulation among other staff, care home residents and their family members. The invitation email included a summary of the inclusion–exclusion criteria to aid staff in identifying potential participants. These documents were written in English and translated into Chinese language.

The inclusion criteria were Chinese residents who lived in the selected care homes during COVID-19 lockdowns, family members who were a relative of such a Chinese care home resident, and staff participants involved in the care of Chinese residents during the lockdown periods. We included participants fluent in Mandarin Chinese or English. This study excluded Cantonese Chinese speakers, because the main research interviewer (DZ) spoke Mandarin Chinese. Residents were also excluded if they were unable to give consent or undergo interviews because of significant sensory deficits, cognitive impairment or physical limitations. Ability to give informed consent and participate in qualitative interviews was assisted by talking to staff members who were familiar with the care home residents. Bedside cognitive testing was not conducted with the care home residents. Care home managers confirmed the availability of potential participants at their facilities and arranged times for interviewing to proceed. The demographics of the two care homes recruited into this project are shown in Table 1.

**Table 1.** A summary of care home demographics.

	Care home A	Care home B
Ethnicity of owner	Chinese	Chinese
Level of care	Dementia care^a^	Rest home^b^ and hospital care^c^
Number of beds	29	58 in total: 18 at rest home level care, 40 at hospital level care
Approximate number of Chinese residents (during the lockdown periods)	3	Majority Chinese
Examples of personnel available	Registered nurse, healthcare assistants, physiotherapist, diversional therapist, activities coordinator, dietitian, chaplain	Registered nurse, healthcare assistants, physiotherapist, diversional therapist, activities coordinator, dietitian, chaplain
Level of security	Digital lock on the front gate	Digital lock on the front gate
Examples of activities available	Art, bingo, board games, pet therapy, entertainment (music, performances), exercises, outings, pastoral care, singing	Art, bingo, board games, pet therapy, entertainment (music, performances), piano, exercises, outings, shopping, pastoral care, singing
Garden	Yes	Yes
Chinese staff available (Mandarin or Cantonese)	Yes, but most staff cannot speak Chinese	Yes, most staff can speak Chinese
Details about meals	Mix of Chinese and European style cuisine	Mix of Chinese and European style cuisine

^a^ For individuals with dementia who have safety concerns or possible behavioural issues. Usually have higher levels of security than a typical care home.

^b^ For older adults who are largely independent or only require some assistance with personal care and general day-to-day activities. Many have some degree of cognitive impairment.

^c^ For older adults with significant disability or medical concerns. Most individuals require the assistance of up to two people for daily activities.

Informed consent was obtained from participants. We recruited participants from two care homes: care home residents (*n* = 6), family members (*n* = 6) and staff (*n* = 6). The interviews were conducted using a semi-structured topic guide. This contained questions translated in both English and Chinese (see Appendices 1–3 available online for the English version of each topic guide).

### Data collection

We conducted face-to-face interviews with care home residents, family members and staff (DZ). These interviews were conducted in English or Mandarin and were audio-recorded. Field notes were taken immediately after each interview, documenting the location of the interview, a brief description of the interviewee and reflections about the interview process, such as interruptions, changes in interview location and interviewer preconceptions.

### Transcribing of interviews

The audio-recordings of the interviews were transcribed verbatim by three transcribers, including the first author. Every transcript was checked against their respective audio-recording. Participants who expressed interest in checking their completed transcript were sent a password-encrypted file via email along with scanned consent forms. Revisions were asked to be made within ten days. Once revisions were made and identifying features were removed, finalised transcripts were uploaded to NVivo 12, a qualitative data analysis software, for coding.

### Coding and thematic analysis

The transcripts were coded initially in their original language without translation (DZ, English or Chinese). Coding was completed (DZ) using NVivo 12 before constructing a coding framework through discussion with the other co-authors. Themes were synthesised and modified by consensus among the research group: two academic old age psychiatrists and an academic adult psychiatrist. DZ, GC and LN are of Chinese descent and offered a Chinese perspective to the interpretation of data. SC previously resided in Malaysia and Singapore for two years and had prior knowledge of Chinese culture. DZ and GC are bilingual and able to read simplified Chinese script. Therefore, they were able to code both English and Chinese transcripts in their original text. LN and SC co-coded English transcripts.

## Results

There were 18 participants in total: 6 residents (R), 6 family members (F) and 6 staff members (S) (Tables 2–4). Care home A was a dementia unit. Three Chinese residents all had severe dementia and therefore were excluded from the study. Care home B was a care home where Chinese residents and staff were in the majority. The family members interviewed were not related to the resident participants. Interviews ranged from 20 to 60 minutes. Locations included the staff office area in care home A, the common lounge area of care home B and private resident rooms in either care home.

**Table 2.** A summary of resident participant demographics.

Participant	Age	Sex	Country of origin	No. years in New Zealand	No. years in care home	Marital status	No. of children	Usual visitor	Care home A/B	Interview language
R1	79	M	Mainland China	13	2	Married	2	Wife	B	Mandarin Chinese
R2	91	F	Mainland China	25	6	Widowed	3	Son and friend	B	Mandarin Chinese
R3	76	F	Mainland China	1	1	Widowed	1	Son	B	Mandarin Chinese
R4	79	M	Hong Kong	26	2	Married	1	Wife and son	B	English
R5	66	F	Mainland China	6	1	Married	2	Daughter	B	Mandarin Chinese
R6	83	F	Mainland China	20	3	Widowed	2	Son and daughter	B	Mandarin Chinese

M = male; F** **= female.

**Table 3.** A summary of family participant demographics.

Participant	Age	Sex	Country of origin	No. years in New Zealand	No. years family members have been in the care home	Usual no. of visits per week	Relationship to resident	Care home A/B	Interview language
F1	52	F	Mainland China	10	1	2	Daughter	A	Mandarin Chinese
F2	86	F	Mainland China	24	0.5	3	Wife	B	Mandarin Chinese
F3	73	F	Mainland China	2	1	Daily	Wife	B	Mandarin Chinese
F4	71	F	Mainland China	3	3	Daily	Wife	B	Mandarin Chinese
F5	70	F	Mainland China	6	5	Daily	Wife	B	Mandarin Chinese
F6	57	F	Mainland China	29	2	4	Daughter	B	Mandarin Chinese

M = male; F = female.

**Table 4.** A summary of staff participant demographics.

Participant	Sex	Country of origin	Role	No. years at the care home	Care home A/B	Interview language
S1	F	South Africa	Care home manager and clinical manager	5	A	English
S2	F	Mainland China	Registered nurse and owner	6	A	English
S3	F	Mainland China	Care coordinator	4	B	Mandarin Chinese
S4	F	Mainland China	Care coordinator	8	B	Mandarin Chinese
S5	M	Mainland China	Health care assistant	5	B	Mandarin Chinese
S6	F	Mainland China	Activity coordinator	3	B	Mandarin Chinese

M = male; F = female.

The transcripts were sent to five participants who expressed interest in receiving them via a password-encrypted file. We constructed a coding framework, generated a thematic map. Themes were refined after robust discussion between the coding team (Braun & Clarke, 2021).

### Themes

**Figure 1.**
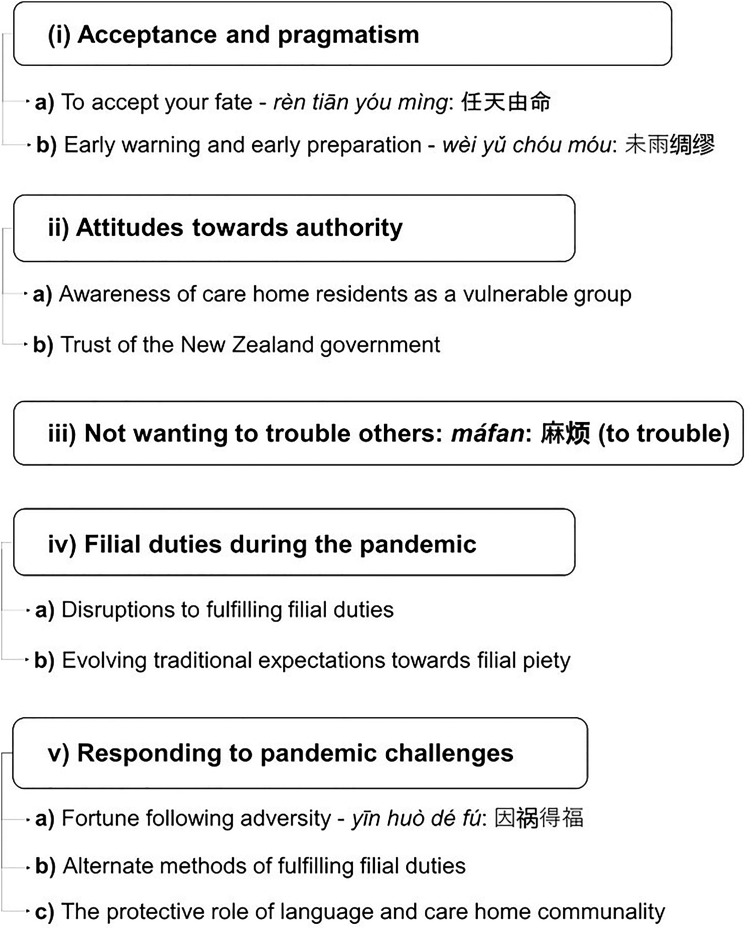
Experiences of New Zealand Chinese older adults in care homes during lockdowns: main themes.

Five themes were identified: (a) acceptance and pragmatism, (b) attitudes towards authority, (c) the concept of *máfan*: 麻烦 (to trouble), (d) challenges to fulfilling filial duties, and (e) responding to pandemic challenges (Figure 1).

#### Acceptance and pragmatism

##### To accept your fate – *rèn tiān yóu mìng*: 任天由命

Several participants reported acceptance of what was beyond their control during the lockdowns, illustrating *rèn tiān yóu mìng*: 任天由命, meaning ‘to accept your fate as determined by the heavens’. This quote originates from a Confucian proverb, *shēng sǐ yǒu mìng, fù guì zài tiān*: 生死有命，富贵在天, meaning ‘life and death are determined by fate, wealth and prosperity are determined by the heavens’. The next line in the original text is, *jūn zǐ jìng ér wú shī, yŭ rén gōng ér yǒu lǐ, sì hǎi zhī nèi, jiē xiōng dì yě*; 君子敬而无失, 与人恭而有礼, 四海之内, 皆兄弟也, which translates to, ‘If you uphold your noble character and respect towards others, you will have family all over the world’. It is framed as inspirational guidance for individuals to accept what cannot be changed and to uphold one's morals in the face of adversity which may equate to participants’ pragmatic acceptance of pandemic conditions:

The emotional impact of things? What impact, just *rèn tiān yóu mìng*: 任天由命, if you get infected then it can’t be helped, but if you don’t go out then you won’t get infected. So, I just don’t go anywhere. (R3)

Participants shifted their focus from what was beyond their control to what they could influence:

I'm not worried because I think the pandemic is more of a large-scale than a personal issue. No one likes this virus, but since it's here, everyone needs to cooperate. If the government says don’t go out, then don’t go out. We should follow these rules. (F5)

They accepted that they could not determine when the lockdowns would conclude but could abide by infection measures to minimise the spread of the virus. Some avoided lingering on negative emotions related to visiting restrictions.

##### Early warning and early preparation – *wèi yŭ chóu móu*: 未雨绸缪

This theme reflects a Chinese attitude of exercising caution and preparing for the worst-case scenario as per the idiom *wèi yŭ chóu móu*: 未雨绸缪, meaning ‘to wrap your house in silk before the rain’:

How should I say this, us Chinese usually *wèi yŭ chóu móu*: 未雨绸缪. If we know there will be some challenge in the near future, then we’ll prepare and buy whatever we need beforehand. (F6)

Some staff participants described preparing and planning protocols after the first COVID-19 outbreak in China:

Because S2 had relatives over there […], we were prepared for COVID coming here, and we were also warned that we would need to look after our own residents. […] So, we knew exactly when we were locking down and what was happening. (S1)

A heightened awareness of the virus hastened early preparation due to participants’ connections to relatives overseas and Chinese media:

China was the first to have a lockdown, so we were emotionally prepared. (F6)

#### Attitudes towards authority

##### Awareness of care home residents as a vulnerable group

Resident participants considered themselves a vulnerable group and therefore accepted more intensive infection measures:

If we protect the care home, then we protect the old. Because our old people are very vulnerable, what if they can’t take the infection? Some can’t even afford a common cold, let alone COVID. So, we have to take extra care to protect them. (S3)

In general, Chinese participants expressed a sense of duty and obligation in abiding by infection measures as good citizens of New Zealand society:

These are rules, and you have to follow them. They’re the care home's rules, and you can’t just randomly leave the home because you’re old –it's not worth it. (R3)

Residents trusted staff working at care facilities:

They closed the doors, and we couldn’t go out. We lost our freedom. But closing the doors was for our own safety, so it couldn’t be helped. If the doors stayed open, anyone could come in, and you’d have no idea if they were infected, so I think it was a very effective measure. We were safer this way. (R6)

Some residents spoke negatively of the restrictions, describing them as a ‘jail’ or ‘concentration camp’. Others reported that they felt safe within their facilities. Visiting restrictions and the use of personal protective equipment mitigated their sense of fear about a possible outbreak. Several described the care home as a ‘safe haven’.

##### Trust of the New Zealand government

Some participants wanted to follow rules and obey the government as community obligation:

Everybody feels lonely, lockdown at home and cannot reach the outer world. But this is a must […], and you just say that Jacinda [Prime Minister] said, keep the rules and stay home and remain isolated when you don’t feel well. You don’t transmit the disease to others, it's happier for you and happier for the family and happier for the community. (R4)

Many expressed gratitude to the New Zealand government for their decision-making and support:

The government is doing well. Anyone would think so. We should understand them […] China is good as well, but the New Zealand government seems to think about us a bit more […] for example, they’ll give us benefits and other support. (R3)

Many were reassured that the government was protecting its citizens:

We trust the government, so we weren’t too worried at the time because we had that sense of trust there. We trusted that the government would get things under control. (F6)

The success of the New Zealand government in eliminating the COVID-19 virus during the 2020 lockdowns reinforced this sense of trust. Participants felt fortunate to be living in New Zealand and compared their circumstances with others living overseas:

New Zealand is a lucky place. People got protected. Actually, it's the best performance in the world where people don’t get the virus easily. You see, New Zealand and Taiwan are the two places where you have good control, good government help, it means less people suffer from the disease. Not like other countries, you see? (R4)

Participants reported they were willing to follow regulations because they viewed the government as a uniting force for New Zealand. They trusted that staff, as figures of authority, were acting in the best interests of the residents.

#### Not wanting to trouble others – *máfan*: 麻烦 (to trouble)

Several resident participants expressed a reluctance to *máfan*: 麻烦, or ‘to trouble’ staff and family members during the lockdown periods:

I miss them, but what's the use of that? They’re busy with work. They have two kids that need to go to school, and they’re busy. It can’t be helped. I don’t want to bother them. (R3)

Residents observed that family members had other responsibilities and did not wish to prioritise their own needs ahead of their family and staff:

Because I’m relatively healthy, I don’t interact with them as much. But I see them helping others get up from bed and eat, the staff have it really hard. […] With the pandemic, they have to wear masks as well, which is even more difficult for them. (R6)

Residents stated that they tried to be self-sufficient, only asking for additional help if needed. Family members were aware of increased pressures at the care home and wanted to make things easier for staff:

Sometimes I need something done, but I’m worried that I’d make things harder for staff. During lockdown, family members can’t come and help, so they were already under strain. They took photos of every single resident during lunchtime every day. (F4)

Some residents expressed a degree of shame in relying on others. *Diū liǎn*: 丢脸, or ‘losing face’, reflecting concerns by family members if residents were to misbehave:

I’m most worried about him making a scene, because sometimes he does that when I don’t visit. […] So many staff have to take time out to comfort him. […] It causes trouble for the staff, and sometimes when he's eating and misbehaves, he spills food onto people's clothes. […] This place is 70 per cent Chinese residents, so it's quite nice. I don’t want him to misbehave, or else we’ll have to go up a level of care. (F2)

Family members reported a sense of helplessness in not being able to visit or help their family members and concerns that their relative might cause trouble for the staff.

#### Filial duties during the pandemic

##### Disruptions to fulfilling filial duties

Some residents became distressed as face-to-face contact between residents and family members was limited, despite being a key component of fulfilling filial duties. Visits were allowed when restrictions eased but were limited to short, socially distanced periods, at a specific location:

It was hard to only have one hour with him. I’d bring some food, fruits, biscuits, set them out for him, […] and then half an hour would be already over. (F2)

Participants experienced feelings of anxiety during the lockdown, a sentiment illustrated by *cǎo mù jiē bīng*: 草木皆兵, a Chinese idiom meaning ‘to see every tree and bush as an enemy soldier’. It refers to a feeling of fear about an impending threat. There was an undercurrent of unease within the care home environment with fear of infection spreading from staff who traversed boundaries between the care home and the outside world:

During the first lockdown, everyone was *cǎo mù jiē bīng:* 草木皆兵. (S6)

Family members often felt time-pressured during these visits and struggled to engage with their relatives. Many chose not to visit at all during higher alert levels. There were changes in how residents perceived the outside world, because it was perceived as a place of danger. This fear of the outside world was also apparent among staff:

We just felt so… Threatened by people on the other side of the fence. (S1)

Some residents began to view family members as the vulnerable ones and themselves as the protectors. They became worried about the safety of their families outside the care home:

I told my daughter, “You have to be careful.” I told them not to take the kids to places with more people. […] For us, we don't go out, so it's okay, but they still need to go shopping, go to work, go to school, so I’m worried that they might catch the virus. (R5)

##### Evolving traditional expectations towards filial piety

Many participants acknowledged that filial duties were disrupted because of unprecedented circumstances and adjusted their expectations. The pandemic was described as *tōng tiān zhī nàn*: 通天之难, a general idiom which translates to ‘a great calamity from the heavens’. Participants used this cultural expression to describe their perceptions of the sheer scale of the pandemic and related anxiety. There was a stoic acceptance of conditions imposed, even as traditional filial expectations were defied:

I understand, I really do. I can’t blame my children or blame them for not fulfilling their filial obligations. Though it's true that it's an expectation in Chinese culture, I don’t mind. (R3)

#### Responding to pandemic challenges

##### Fortune following adversity – *yīn huò dé fú*: 因祸得福

Some participants discovered unexpected positive impacts on residents. Staff participants reported that infections were less prevalent during the lockdown periods:

Everyone was healthy and well during that time. Because everyone had been so careful with washing their hands, physical distancing, hygiene, masks… Actually, maybe it's *yīn huò dé fú*: 因祸得福. (S4)

*Yīn huò dé fú*: 因祸得福 a Chinese idiom that translates to, ‘gain a blessing through adversity’ may reflect positive outcomes for some care home residents during the lockdown. Paradoxically, some staff participants reported that some residents had better appetites in the absence of their family members. Some residents also appreciated a greater degree of freedom, autonomy and independence:

[A resident's] wife would come to every lunchtime so that she could help him eat, and then she wouldn’t let him sit up. She forced him to lie down, and then, choking and carrying on, and he wouldn’t eat himself. And once we made him come through to the dining room and sit on a chair and be amongst other people eating, he feeds himself now. (S1)

There were some long-term changes in care home practices as one care home decided not to allow family visitors during mealtimes to minimise distractions.

##### Alternate methods of fulfilling filial duties

Participants adapted to conditions by alternative means. One participant met her relative across the care home fence. These physically distanced visits reassured both family members and residents by allowing them to see each other and communicate in-person briefly:

I wasn’t allowed to visit during the first lockdown. To get around this, I would tell them [the staff] to move him out into the garden behind the fence, so there was some distance between us. I’d be on the outside of the fence, and he’d be on the inside. (F4)

Family members demonstrated filial piety through acts of affection, such as gifts of food or daily essentials. Many would leave items at the entrances of the facilities for residents, which staff members delivered. These gifts acted as temporary placeholders for family members who were separated from residents in the outside world:

In the latter half of lockdown, some family members would leave food at the entrance every day. Staff would go pick it up, and when the residents ate those homemade meals, their appetite improved. (S6)

The gift of food held both symbolic and practical significance. Within Chinese culture, the role of food extends beyond a simple means of sustenance to be a symbol of respect, affection, and love. Food was a reminder of their family, who had cooked the meal themselves. Family members delivered other items:

My son helped me buy masks and the such. He said that you should wear a mask if you need to go outside. (R2)

The gifting of masks was an attempt to protect their relative inside the care home from infection during the pandemic. Technology was an alternate means to fulfil filial duties. The use of video calls through a Chinese social media application, WeChat, mitigated residents’ feelings of loneliness:

It resolved my feelings of wanting to see them. If I think of them, then I can just use WeChat. (R6)

Staff observed that residents would smile more and seem more relaxed after video calls.

At care home B, several family members were given the option to live with their relative at the care home throughout the lockdown periods. They became part of the residential lockdown bubble. Family members did not need to pay for their length of stay and were treated as residents during this time. Many of these participants cited a sense of duty to take care of relatives:

They told me if I went home, I wouldn’t be able to visit. So, in order to take care of my husband, I decided to stay. I stayed throughout all the lockdowns. (F5)

##### The protective role of language and care home communality

The Chinese language is entwined with Chinese culture, not a separate or distinct entity. The Chinese-speaking environment connected residents to their heritage, as reflected by the following idioms:

*hù xiāng lǐ jiě*: 互相理解, which translates to ‘understand each other’;

*hù xiāng pèi hé*: 互相配合, ‘cooperate with each other’;

*hù xiāng bang zhù*: 互相帮助, ‘help each other’.

The recurring phrase *hù xiāng*: 互相 means ‘mutual’, implying an intimate, two-way connection between members of the group. These phrases reflect a collective approach to lockdown conditions. During the lockdown, each care home became its own ‘bubble’ akin to a surrogate family for the residents. Residents were able to interact and socialise with each other:

There are a lot of Chinese people here. […] We have seven rooms, […] five are occupied by Chinese –some from Taiwan, some from the mainland. I have lots to chat about with them. (R3)

The shared Chinese language helped the care home residents connect with each other, especially non-English-speaking residents:

Sometimes when the care staff come into his room, if they’re Chinese, then he can chat with them a little, yeah, chat with them a bit more. […] If they aren’t Chinese, he can’t understand them. (F4)

The language contributed to familiarity and belonging within the facility. Care home A was a mainly English-speaking care home, and the lack of a Chinese-speaking environment may have exacerbated feelings of loneliness for some.

Having Chinese-speaking staff gave residents and family some comfort:

I don’t think it [not knowing English] affected him. Because there are Mandarin speakers here, Cantonese speakers, also English speakers. If he can’t explain things well, they’ll call for staff that know his language. (F6)

Communication between staff and residents was not limited to verbal language; non-verbal cues were acknowledged to be important. Staff at care home A cared for residents with severe dementia and were familiar with non-verbal cues in interactions.

## Discussion

The findings of this study reflect the unique cultural lens through which our participants experienced lockdowns and how Chinese attitudes and values influenced a time of adversity. Findings included themes of acceptance and pragmatism, attitudes towards authority, not wanting to trouble others (*máfan*): 麻烦, challenges to fulfilling filial duties, and responding to pandemic challenges.

*Rèn tiān yóu mìn*g: 任天由命, to ‘accept whatever comes’ reflects a Chinese approach to facing hardships in life, in which fate is viewed as an immutable, unconquerable force. Only the heavens can decide, and one should find peace by setting their sights on what they can control or how they act within their position in society. The care home residents accepted what was beyond their control and focused on what they could do. Many adopted a pragmatic approach and focused on minimising infection risk. Accepting one's fate has been observed among Chinese older adults in the United States (Dong et al., 2011). This reflects Chinese philosophical values with similarities to concepts found in mindfulness or other acceptance-based therapies. Although acceptance is also practised in other cultures, the concept is often tied to the individual and linked to growth in character – for example, in Christianity, acceptance is seen to build character through overcoming hardship in accordance to the will of God (Xie & Wong, 2021). On the other hand, *yīn huò dé fú*: 因祸得福, to ‘gain a blessing through adversity’ has a different nuance to *rèn tiān yóu mìn*g: 任天由命. The saying *yīn huò dé fú*: 因祸得福 is similar to another traditional proverb, *sài wēng shī mǎ*; 塞翁失马, which means that when something bad happens, it may turn out to be a blessing. Contrasting with fully accepting one's fate, this mindset involves reappraising an individual's perspective to focusing on the positives and avoiding dwelling on negative thoughts.

Participants held figures of authority in high regard and felt that it was their duty to obey the rules. These figures included the government, police and care home staff. From a Confucian perspective, the government is analogous to a familial structure in the context of a broader societal hierarchy (Ng, 2000). The central government is seen as a father figure, unrelenting and demanding utter obedience from his children. As his children, the general public is expected to fulfil their duty of obeying their government.

Participants in this study may have interpreted restrictions during the pandemic with a collective sense of utilitarianism. The decisions to abide by and be compliant with government decree may stem from Confucian rules but may also reflect mutual inclination to follow rules. Many agreed that restrictions were necessary for the safety of the collective. The Chinese identity is strongly related to relationships to others, and self-sacrifice for the benefit of the collective is in keeping with this. The Confucian perspective views authority as paternalistic and may explain why Chinese people are more likely to comply with decisions made by figures of authority (Cong, 2004; Ng, 2000). Their decision was also influenced by their understanding of the scientific explanations provided to them during the pandemic. They felt that the health risks of infection outweighed the psychological risks of restrictions and isolation. The pandemic highlighted the divide between individuals who comply with regulations and those who do not. The willingness of participants to follow the rules may have had a protective effect, contributing to cohesion among care home residents. The duality of resident perspectives illustrated this during the lockdowns, where many viewed restrictions as simultaneously a stripping of their freedoms as well as a source of protection and reassurance.

The fear of being a burden to family has been identified among older New Zealand Chinese people. This fear is linked to the Chinese concepts of *máfan*: 麻烦 and *diū liǎn*: 丢脸. For Chinese older adults, independence is an adaptive means to avoid relational conflict and maintain relational harmony between themselves and other family members (Lou & Ng, 2012). This idea of self-sacrifice to minimise *máfan*: 麻烦 (‘trouble’) for the collective's interest is seen as a virtue in Chinese cultures, where the collective is held above the individual. Conversely, causing *máfan*: 麻烦 (‘trouble’) for others within the collective may be a source of shame, or *diū liǎn*: 丢脸 for Chinese individuals. In times of hardship, familial responsibilities and upholding the well-being of collective may mean that Chinese older adults place the needs of other family members above themselves. This collective mindset may exacerbate loneliness (Park et al., 2019). Chinese older adults are reluctant to access services because they do not want to burden the health system (Yeung & Allen, 2020). This mindset may further isolate Chinese older adults from family and health services.

In Chinese culture, filial piety (*xiào*: 孝) is a tradition of respecting parents, elders and ancestors. Residents were faced with emotional conflict in complying with rules and their own expectations of family. In modern China, ‘adult children shall have the obligation to support and assist their parents’, and filial piety is still an expectation among New Zealand Chinese (Chinese Communist Party, 1982; Yeung & Allen, 2020). A lower receipt of filial piety is a risk factor for suicidal ideation among Chinese older adults in western societies (Simon et al., 2014).

The evolution of traditional views towards filial piety in modern society has changed filial behaviour among older New Zealand Chinese immigrants, who may be more relaxed and flexible (Yeung & Allen, 2020). Filial displays of respect have traditionally been practical, such as providing financial support, but have shifted to emotional acts of affection (Li, 2011; Yeh et al., 2013). Many Chinese New Zealanders wish to maintain Chinese traditions and values within western society. The pandemic has provided an impetus to adapt cultural norms, and the concept of filial piety among New Zealand Chinese can be expected to evolve further. When considering services for older Chinese, the fluid nature of filial piety should be acknowledged (Chow, 2006; Lin et al., 2015).

This study has provided examples of how Chinese culture and values are a source of resilience. Older adults fare better than their younger counterparts in terms of mental health impacts related to COVID-19, despite perceptions of frailty and passivity (Every-Palmer et al., 2020; Gasteiger et al., 2021; Morgan et al., 2021; Parlapani et al., 2021). Cultural influences help older adults develop resilience, particularly those who have immigrated (Ong & Bergeman, 2004; Xie & Wong, 2021).

Confucian values were prominent in this study. The well-known idiom, *tóng zhōu gòng jì*; 同舟共济means, ‘to cross the river in the same boat’. There was a sense of connection and community among participants, enhanced by speaking Chinese and using Chinese social media. Social relationships, not limited to familial ones, are an important component of resilience among older Chinese adults and are protective against loneliness (Faure & Fang, 2008; Lai et al., 2020; Lou & Ng, 2012). For Chinese people, social relationships are essential and enhance psychological well-being. This is particularly true for older Chinese, as is their cultural identity, position and connection to society (Li, 2011).

### Strengths and limitations

The inclusion of Chinese participants from a care home where Chinese residents comprise the majority allowed for in-depth exploration of the lockdown experience. The interviews were conducted in-person, which allowed for a more intimate interviewing space where participants could express themselves freely. The first author (DZ) was fluent in both Mandarin Chinese and English and interviewed participants in their preferred language, enabling rapport to be built. Reflexivity was enhanced by taking thorough field notes after each interview. All interview transcripts were double-checked against the original audio file to ensure accuracy. Five interview transcripts were checked by willing participants. The first author transcribed, coded and analysed the data, which provided consistency in data handling. Transcripts were co-coded by three additional coders, two of Chinese descent who offered a further cultural perspective to the interpretation of data. This led to nuanced discussions regarding the data. The three groups of interviewees were triangulated, which contrasted experiences.

There are several limitations, however. The sample size for each group was relatively small. The majority of interviews were conducted in care home B, where most residents and staff were of Chinese ethnicity. As noted in the results, people with severe dementia were excluded from the study. One limitation is that we did not include any Chinese residents with severe dementia in care home A. Therefore, residents’ perspectives are limited to care home B and quotations provided by staff members provide just one perspective from that care home. The majority of participants originated from Mainland China, and Cantonese-only speakers were not included in the study, despite 21.3% of Chinese in New Zealand being Cantonese speakers (Statistics New Zealand, 2019). For this reason, the participants may not be representative of the overall demographic of Chinese residents in New Zealand care homes. The New Zealand setting may limit transferability of findings to international contexts. The interview covered three lockdown periods as opposed to a single lockdown; there may have been recall bias in the various experiences of restrictions.

## Recommendations for practice and directions for future research

The pandemic experiences of resident participants were underpinned by Chinese culture and philosophy, which may shape the experiences of care home residents in other stressful life situations. There are guidelines for culturally and linguistically diverse peoples with dementia but there is a lack of research into the specific needs of ethnic subgroups. This has resulted in recommendations for a heterogeneous population of older adults in New Zealand that do not take into account their ethnic differences (Peri & Cheung, 2016). There is a call for more contextualised research that acknowledges the roles of culture and societal influences rather than a ‘one size fits all’ approach (Brayne & Miller, 2017; Roche et al., 2021). We consider it necessary for health professionals, and organisations, to understand Chinese culture and philosophy when providing healthcare for older Chinese.

The importance of family and relationships was emphasised by our study participants. Our findings suggest that future interventions should focus on building relationships between Chinese care home residents and their wider community. This may include facilitating community events, group activities or Chinese customs which are social in nature, such as communal eating or the sharing of Chinese tea. These cultural practices were disrupted in the care home environment during the lockdown. The collective attitude towards authority identified in this study indicates that clear protocol and communication are effective in unifying Chinese care home residents and their families in planning for future pandemic scenarios.

Older Chinese immigrants may benefit from peer-based interventions involving individuals from similar Chinese cultural backgrounds (Lai et al., 2020). Our study has shown that an environment which caters towards Chinese culture and language is both enriching and protective during times of adversity. Technology and social media are an effective means of maintaining relationships in the care home environment, particularly when the usage is facilitated by staff (Zhang et al., 2022). Our study demonstrates that the presence of staff from a similar cultural background is helpful for residents. Understanding Chinese perspectives could enable staff to develop awareness and attune to the needs of ethnic minority residents in care homes.

In considering other cultures, we make the following recommendations: educating staff to be sensitive to the needs of specific ethnic groups; training or written resources for workers caring for ethnic minority care home residents; and, investing in digital literacy training for employees at care home facilities to assist older persons and staff to use technology. In future lockdown scenarios, we recommend care home flexibility with restrictions to allow the delivery of homemade food or other goods.

## Conclusion

The pandemic disrupted the fulfilment of filial obligations during visiting restrictions, a key component of Chinese culture. Key sources of resilience among Chinese care home residents were the maintenance of relationships and connections within the care home community. We recommend culturally specific guidelines, tailored to the needs of minority groups within care homes. This study presents the lockdown through a uniquely Chinese lens. Our findings may contribute to a specific guideline for Chinese older adults in residential care, in planning for a similar pandemic scenario in the future. It may also set a precedent for future research into the needs of other minority groups.

## Supplemental Material

sj-docx-1-tps-10.1177_13634615241296310 - Supplemental material for Lockdown through a Chinese lens: A qualitative study Supplemental material, sj-docx-1-tps-10.1177_13634615241296310 for Lockdown through a Chinese lens: A qualitative study by Doris Zhang, Gary Cheung, Sarah Cullum and Lillian Ng in Transcultural Psychiatry

sj-docx-2-tps-10.1177_13634615241296310 - Supplemental material for Lockdown through a Chinese lens: A qualitative study Supplemental material, sj-docx-2-tps-10.1177_13634615241296310 for Lockdown through a Chinese lens: A qualitative study by Doris Zhang, Gary Cheung, Sarah Cullum and Lillian Ng in Transcultural Psychiatry

sj-docx-3-tps-10.1177_13634615241296310 - Supplemental material for Lockdown through a Chinese lens: A qualitative study Supplemental material, sj-docx-3-tps-10.1177_13634615241296310 for Lockdown through a Chinese lens: A qualitative study by Doris Zhang, Gary Cheung, Sarah Cullum and Lillian Ng in Transcultural Psychiatry
