# Supplementary material for: Lockdown through a Chinese lens: A qualitative study
Source: Transcult Psychiatry. 2025 Jan 29;62(2):214–26. doi: 10.1177/13634615241296310 (PMC12130599; doi:10.1177/13634615241296310)
Supplement: sj-docx-1-tps-10.1177_13634615241296310 - Supplemental material for Lockdown through a Chinese lens: A qualitative study [file sj-docx-1-tps-10.1177_13634615241296310.docx]

Appendix 1: Resident participant topic guide

Resident demographics: Age, sex, country of origin, how long they have been in New Zealand for, marital status, number of children, how long they have lived in the facility for, who usually comes to visit them.

1. What was living in the facility during the quarantine and lockdown period like for you? How did this differ from before?
2. I understand that during the lockdown period, you hadn’t seen your family for some time. What was it like not being able to have visitors from family and friends? Did you experience loneliness during lockdown?
3. What did you do to cope with this? (Did you use any forms of technology including telephone to keep in contact with your family during lockdown? How did you find using these technology)
4. What changes in care in the rest home did you notice during the lockdown? (If staff were using full PPE,) what was it like seeing staff coming into your room with full PPE?
5. What information were you given about (COVID-19 and) the lockdown? Did you understand what was going on? Did you receive any written information (in your language)? (If there was a language barrier,) did the language barrier affect your experience? In what way?
6. Has your physical or emotional wellbeing changed as a result of being in lockdown? In what way? Can you be more specific?
7. What did you miss the most during this period?
8. What support or specific measures would have helped you to cope better with the lockdown?
9. As a Chinese person, were you treated any differently during the lockdown? Did you experience any discrimination of any kind from others? (If yes, who and what specifically? Can you give me an example?)
