# Supplementary material for: Lockdown through a Chinese lens: A qualitative study
Source: Transcult Psychiatry. 2025 Jan 29;62(2):214–26. doi: 10.1177/13634615241296310 (PMC12130599; doi:10.1177/13634615241296310)
Supplement: sj-docx-2-tps-10.1177_13634615241296310 - Supplemental material for Lockdown through a Chinese lens: A qualitative study [file sj-docx-2-tps-10.1177_13634615241296310.docx]

Appendix 2: Family participant topic guide

Family demographics: Relation to resident, age, sex, marital status, country of origin, how long they have been in New Zealand for, how long their relative has lived in the facility for, how often do they visit their relative.

1. The facility went into lock down for three periods in the last year, preventing family to visit. How did this make you feel? What were you most concerned about?
2. How did you stay engaged with your family member during this time?
3. What do you think the impact of this restriction has been on your family member? Did they experience loneliness during lockdown?
4. Do you understand what lockdown or quarantine meant? What were you most concerned about?
5. What was the impact of this on you and your family?
6. (If there was a language barrier,) did the language barrier affect your experience? In what way?
7. What do you think the impact may have been on your family member in the facility?
8. What do you think was helpful for your family member during this time?
9. Did you use any forms of technology including telephone to keep in contact with your relative during lockdown? How did you find using these technology?
