# Supplementary material for: Lockdown through a Chinese lens: A qualitative study
Source: Transcult Psychiatry. 2025 Jan 29;62(2):214–26. doi: 10.1177/13634615241296310 (PMC12130599; doi:10.1177/13634615241296310)
Supplement: sj-docx-3-tps-10.1177_13634615241296310 - Supplemental material for Lockdown through a Chinese lens: A qualitative study [file sj-docx-3-tps-10.1177_13634615241296310.docx]

Appendix 3: Staff participant topic guide

Staff demographics: Role, how long they have been working at the facility, age, gender, ethnicity, country of origin.

1. Visiting restrictions were implemented in the facility during the lockdown period of last year.  This meant that residents were no longer able to have face to face visits from family or friends. What was the impact on residents? Can you give examples? Did they experience loneliness during lockdown?
2. What did staff do to encourage ongoing interaction with family and friends? What is the role of technology, including telephone?
3. How did this help residents to stage engaged with family? What were the barriers? What were the benefits?
4. If quarantine was implemented: (Quarantine of all residents occurred on 10^th^ April. This mean they were kept in their rooms and were unable to interact with other residents. What was the impact of this on residents? Can you give examples?)
5. If staff wore PPE: (How did residents respond to staff wearing PPE?)
6. Have you noticed a change in resident’s status during and after the lockdown period? Consider:
7. Emotionally (mental health)
8. Physically (eating, drinking, other)
9. Level of function (ADL’s, mobility)
10. Socialisation
11. Cognitive function
12. Behaviour (particularly residents with dementia)
